# Is there selective retroactive memory enhancement in humans?: a meta-analysis

**DOI:** 10.3758/s13423-023-02372-5

**Published:** 2023-09-25

**Authors:** Damian Koevoet, Albert Postma

**Affiliations:** Experimental Psychology, Helmholtz Institute, Utrecht University, Utrecht, The Netherlands

**Keywords:** Adaptive memory, Selective retroactive memory enhancement, Meta-analysis, Behavioral tagging

## Abstract

Memory is an adaptive and flexible system that preferentially stores motivationally relevant information. However, in some cases information that is initially irrelevant can become relevant at a later time. The question arises whether and to what extent the memory system can retroactively boost memories of the initially irrelevant information. Experimental studies in animals and humans have provided evidence for such retroactive memory boosting. Additionally, these studies suggest that retroactive memory enhancement (RME) can be selective to the semantic meaning of the material. Nonetheless, recent experimental work could not replicate these findings, posing the question whether the selective RME effect is reliable. To synthesize the available evidence, we conducted meta-analyses of 14 experiments. Although the classical meta-analytic procedure suggested a small selective RME effect, Cohen’s *d*_z_ = 0.16, when accounting for small-study bias using robust Bayesian meta-analysis the null hypothesis was supported, Cohen’s *d*_z_ = 0.02, BF_01_ = 3.03. Furthermore strong evidence was found for a bias due to small-study effects, BF_10_ = 11.39. Together, this calls the reliability of a selective RME effect into question.

**Supplementary Information:**

The online version contains supplementary material available at 10.3758/s13423-023-02372-5.

## Introduction

Memory is often viewed as a flexible and adaptive system (Cowan et al., 2021; Kroes & Fernández, 2012; Nairne & Pandeirada, 2008; Nairne et al., 2007, 2008; Ritchey et al., 2016). For example, the theory of adaptive memory posits that motivationally significant events should be prioritized when determining which memories are stored (Nairne & Pandeirada, 2008; Nairne et al., 2007, 2008; Shohamy & Adcock, 2010). This suggests that events that are relevant to one’s survival are preferentially stored over events that are not relevant for survival—as such a mechanism ultimately prolongs life (Nairne & Pandeirada, 2008). However, some events that may initially not seem relevant to our survival, can become more relevant at a later time (Cowan et al., 2021; Dunsmoor et al., 2022; Frey & Morris, 1997; Kalbe & Schwabe, 2021; Moncada et al., 2015; Redondo & Morris, 2011). The question arises whether and how memory systems can adaptively change how well initially irrelevant events may be remembered when they become relevant for survival at a later time.

The synaptic tag-and-capture and the behavioral tagging hypotheses both suggest that memory systems do have such capabilities (Ballarini et al., 2009; Dunsmoor et al., 2015, 2022; Frey & Morris, 1997; Moncada et al., 2015; Redondo & Morris, 2011; Viola et al., 2014). The synaptic tag-and-capture hypothesis, grounded in electrophysiological studies in brain slices, proposes that the storage of memories can be influenced not only by what occurs before but also by what occurs after encoding (Frey & Morris, 1997; Redondo & Morris, 2011). Relatively weak electrical stimulation can set a “tag.” Without reinforcement, this tag will fade, and lasting connections between neurons will not be established. However, if a tag is later supplied with plasticity-related products, the initial tag may be reinforced, and a lasting connection will be created. The behavioral tagging hypothesis has extended the cellular principles from the synaptic tag-and-capture hypothesis to learning and memory (Ballarini et al., 2009; Moncada et al., 2015). Initial tags can also be set behaviorally by encoding material relatively weakly. The behavioral tagging hypothesis suggests that such behavioral tags can be reinforced by subsequent salient events, such as electrical shocks, stress, or novelty (Ballarini et al., 2009, 2013; Dunsmoor et al., 2022; Moncada et al., 2015; Ramirez Butavand et al., 2020; Redondo & Morris, 2011; Yonelinas et al., 2011). Reinforcing behavioral tags will ultimately lead to long-term memory trace formation of the initially weakly encoded material. The current paper will focus on retroactive memory enhancement (RME)—how the memory for a neutral event may be enhanced by a salient event that occurs after the encoding of this neutral event.

Behavioral tagging inspired research has also been conducted in humans (Ballarini et al., 2013; Dunsmoor et al., 2015). RME research in humans can generally be further divided into two groups of studies: selective RME studies and general RME studies. General RME studies attempt to induce RME by boosting all neutral material, regardless of semantic stimuli categories (for human general RME studies, see Ballarini et al., 2013; Cahill et al., 2003; Cunningham et al., 2018; Ramirez Butavand et al., 2020; Ritchey et al., 2017; Yonelinas et al., 2011). Most of the general RME studies use stress (i.e., shocks or cold pressor stimulation) or novelty (i.e., new environment or novel lessons in school) as salient events to reinforce previously set tags (Ballarini et al., 2009, 2013; Cahill et al., 2003; Ramirez Butavand et al., 2020; Redondo & Morris, 2011; Ritchey et al., 2017; Yonelinas et al., 2011). While ample studies have already investigated general RME in both humans and rodents, selective RME has been investigated relatively scarcely and evidence for such a specific RME effect remains equivocal (Bréchet et al., 2020; Clewett et al., 2020; Dunsmoor et al., 2015; Hennings et al., 2021; Kalbe & Schwabe, 2021; Oyarzún et al., 2016; Patil et al., 2017). Selective RME studies attempt to induce RME by linking salience to a specific semantic stimulus category. Selective RME remains hard to investigate in animals when compared to general RME due to its category-specific nature. To our knowledge, no selective RME study has yet been conducted in animals. Therefore, we focus on selective RME in humans here.

This paper aims to provide an overview of the current human selective RME literature. Firstly, we provide an overview of the study designs and methodology of selective RME experiments. Secondly, we address the contradictory selective RME findings by conducting meta-analyses in which the current evidence for human selective RME effects is synthesized. Lastly, we discuss the current findings, examine potential statistical and methodological factors that may affect selective RME, and we provide recommendations for future efforts within the field.

## Methodology in human selective RME studies

**Fig. 1:**
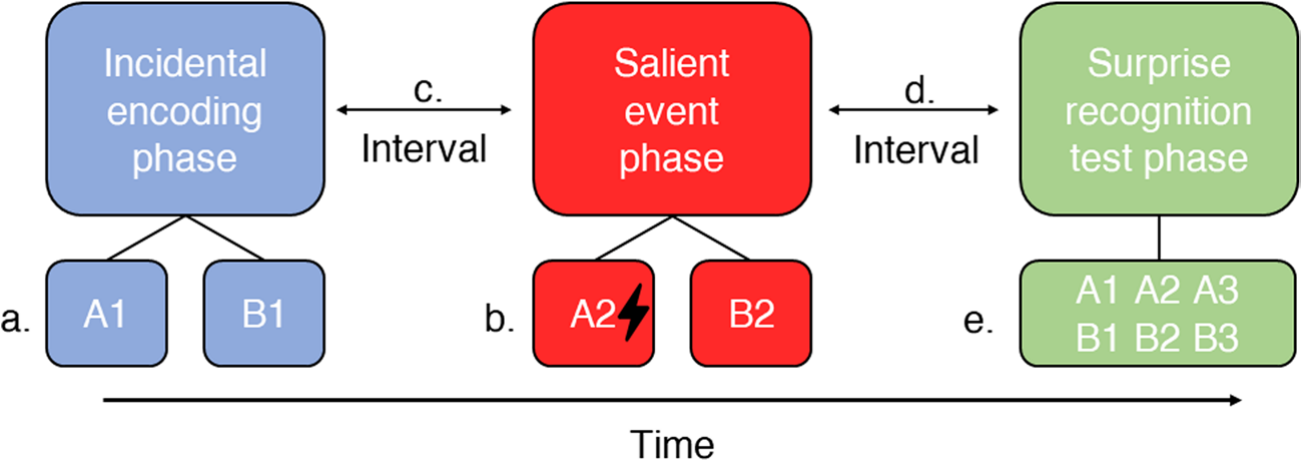
A schematic view of the currently used paradigm in human selective retroactive memory enhancement experiments. **a.** During the incidental encoding phase, neither of the two semantic categories are coupled to a salient event. **b.** During the salient event phase, one of the semantic categories is coupled to a salient event (A2), while the other category is not (B2). The lightning bolt reflects presentation of a salient event. **c.** The interval between the incidental encoding and the salient event phase. **d.** The interval between the salient event phase and the surprise recognition test phase, or consolidation interval. **e.** In the surprise recognition test material is presented from the initial encoding phase (A1, B1), from the salient event phase (A2, B2) and new items belonging to the used semantic categories (A3, B3). In this schematic view, enhanced recognition of A1 items compared with B1 items would indicate selective retroactive memory enhancement

Although there are some notable differences, current selective RME studies in humans thus far use comparable paradigms (for an overview of methods used by each study, see Table S1). These paradigms consist of at least three different phases: the incidental encoding phase, the salient event phase, and the surprise recognition test phase (Fig. 1). It is important to ensure that neutral material is only encoded weakly during the incidental encoding phase, since strong encoding already creates an established (salient) trace (instead of a neutral tag) and any subsequent selective RME effects become undetectable (Dunsmoor et al., 2015, 2022; Redondo & Morris, 2011). Note that some studies include an additional phase after the salient event phase in which neutral material is presented to assess prospective memory enhancement (i.e., how salient events that occur before the neutral event can enhance the memory of the neutral event; e.g., Dunsmoor et al., 2015; Oyarzún et al., 2016). However, prospective memory enhancement is beyond the scope of this paper and deserves its own in-depth discussion.

During the incidental encoding phase, stimuli from two different semantic categories are presented (e.g., animals and tools). In most cases, participants are asked to identify whether stimuli belong to semantic Category A or B (Fig. 1a), but there are also different approaches (i.e., delayed match-to-sample task, see Table S1; Bréchet et al., 2020; Patil et al., 2017). Participants do not know that these items should be remembered for a later memory test—thus, encoding is incidental (Hasher & Zacks, 1979).

During the subsequent salient event phase, stimuli belonging to Category A and B are presented. Importantly, although these stimuli belong to the same semantic category, they are different to the stimuli presented during the incidental encoding phase. Stimuli from either Category A or B are coupled with a salient event (Fig. 1b; often 66,67% of the selected category are coupled with a salient event). The semantic category coupled with a salient event and the category not coupled with a salient event will henceforth be referred as CS+ and CS-, respectively. Examples of salient event couplings are electrical shocks or (monetary) rewards (Clewett et al., 2020; Dunsmoor et al., 2015, 2022; Hennings et al., 2021; Kalbe & Schwabe, 2021; Oyarzún et al., 2016; Patil et al., 2017). Note that a single experiment (Bréchet et al., 2020) used the presence of a body (as opposed to having no body) in virtual reality as a salient event—this is based on work that shows that this manipulation boosts episodic memory through enhanced embodiment/bodily self-consciousness (Blanke, 2012; Blanke & Metzinger, 2009; Bréchet et al., 2019, 2020; Park & Blanke, 2019). During the salient event phase, participants are instructed to identify whether they expect a salient event to occur during CS+/CS− presentation in most studies. Note that this necessitates participants to form explicit expectations for the link between the presented category and salient event which may increase the effectiveness of conditioning. This phase is often followed by a memory test interval of 6–24 hours to allow for the influence of consolidation processes (Fig. 1d; Bréchet et al., 2020; Cowan et al., 2021; Dunsmoor et al., 2015; Patil et al., 2017; Squire et al., 2015)

The last phase consists of a surprise recognition test. At the start of the experiment and onwards participants did not know that they would be tested on their memories during the last phase. During this test, all stimuli presented during the incidental encoding and salient event phases, plus additional nonstudied stimuli are presented. Participants then identify whether they recognize the presented stimuli—often while also providing high/low confidence ratings (Clewett et al., 2020; Dunsmoor et al., 2015; Kalbe & Schwabe, 2021). In order to assess the selective RME effect, recognition scores of CS+ and CS− stimuli that were encoded during the incidental phase are compared. Across studies, recognition scores were calculated in two different ways: corrected recognition scores (proportion of hits minus the proportion of false alarms; Clewett et al., 2020; Dunsmoor et al., 2015; Oyarzún et al., 2016; Patil et al., 2017) or memory sensitivity (*d′* from signal detection theory; Bréchet et al., 2020; Kalbe & Schwabe, 2021). Following the behavioral tagging hypothesis, one would expect a higher recognition score in CS+ stimuli than in CS− stimuli (Dunsmoor et al., 2015; Moncada et al., 2015; Redondo & Morris, 2011) since the initially neutral CS+ items (i.e., tag setting) have afterwards been coupled to a salient event (i.e., production of plasticity-related products). Although study designs are quite consistent throughout the selective RME literature (Table S1; see Discussion), results are varied (Bréchet et al., 2020; Clewett et al., 2020; Dunsmoor et al., 2015; Hennings et al., 2021; Kalbe & Schwabe, 2021; Oyarzún et al., 2016; Patil et al., 2017). To address the inconsistency of selective RME effects in the literature, we conducted meta-analyses to synthesize the findings across all relevant experiments.

## Method

### Literature search

**Fig. 2:**
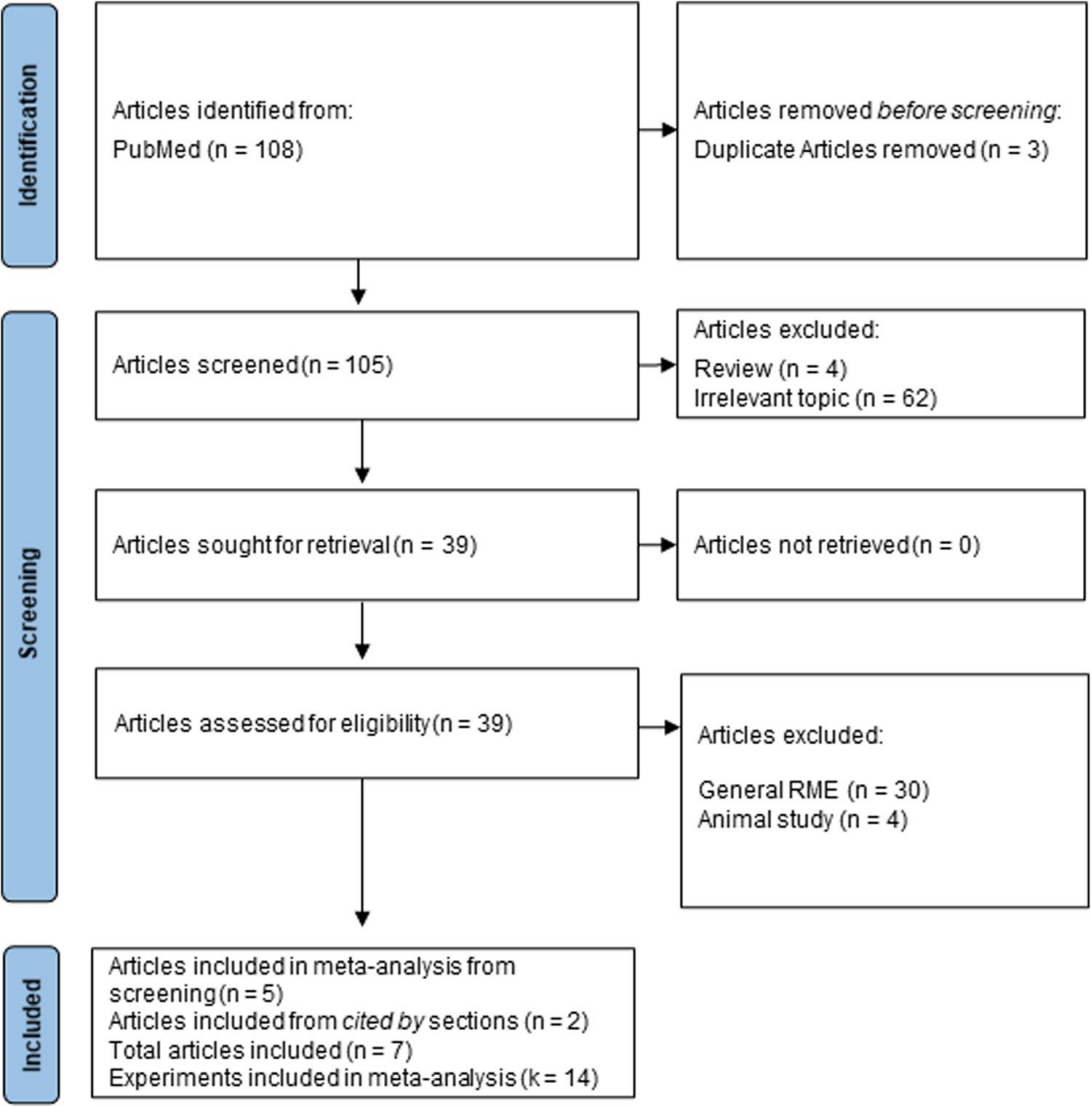
Flowchart of the literature search

The search engine PubMed was used to identify articles that investigated selective RME in healthy humans (Fig. 2). Two searches were performed using *‘retroactive’ AND ‘memory’ AND ‘enhancement’* and *‘post-encoding’ AND ‘memory’ AND ‘enhancement’* as search terms. No age or date restrictions were applied. Articles were only included if they met the inclusion criteria:

- Articles that describe a scientific study using an experimental setup. Meta-analyses and (systematic) reviews were omitted.
- Articles that investigated selective retroactive memory enhancement. Articles investigating general retroactive memory enhancement were excluded.
- Articles that show evidence that the salient event boosted memory accuracy of items presented during the salient event/conditioning phase.
- Articles that investigated human subjects. Data from animal studies were thus omitted.
- Articles were written in English.

The literature search (performed in October 2022) resulted in a total of 105 unique articles (three overlapping articles were removed; Fig. 2). Titles and abstracts of articles were scanned by the first author. Potential articles were read in full to determine eligibility. Next, reference lists and “cited by” sections of included articles were scanned to identify additional relevant articles. Note that no selective RME studies conducted in animals were found. One selective RME study (Hennings et al., 2021) was included in the current meta-analysis, although analyses in the article were collapsed across healthy subjects and subjects experiencing posttraumatic stress disorder symptoms since these groups showed no differences in selective RME (this was the only selective RME study that included a clinical population). Ultimately, seven published articles—consisting of 14 experiments—were included in this analysis.

### Meta-analytic procedure

The goal of the meta-analysis was to synthesize all currently available literature to assess the evidence for selective RME in humans. In this meta-analysis, Cohen’s *d*_z_ (t/**√**n; see Lakens, 2013) was used as the measure of effect size. Cohen’s *d*_z_ is an inherently within-subjects effect size (Lakens, 2013). This is appropriate in the current analysis since thus far selective RME has only been tested using within-subject designs. Consequently, it was unnecessary to adjust the effect size for generalization to between-subjects effects for the current purpose. In sum, we argue that the inherently within-subjects effect size of Cohen’s *d*_z_ is appropriate for the current analyses (Dankel & Loenneke, 2021; Lakens, 2013).

First, a generic inverse variance fixed-effects meta-analysis (Borenstein et al., 2010; Hedges & Olkin, 2014; Hedges & Vevea, 1998) was run using the ‘meta’ package in R (Schwarzer, 2021). A fixed-effects model was chosen since the current experimental designs are similar and there is reason to believe that there is a general underlying selective RME effect across studies (Harrer et al., 2021). Heterogeneity (i.e., a measure of the between-study variance of effect size) was assessed using *I*^2^ and Cochran *Q* tests (Hedges & Olkin, 2014; Higgins et al., 2003) and interpreted using guidelines from Higgins et al. (2021).

To address the possible issue of publication bias (including small-study effects), robust Bayesian meta-analyses were conducted using the ‘RoBMA’ package in R (Bartoš et al., 2022; Maier et al., 2023). These additional analyses were inspired by new developments in meta-analyses and by considerations during the peer-review process. Small-study effects occur whenever experiments with small sample sizes show large effect sizes. Such small-study effects can inflate effect sizes, which can lead to false-positive results (see Gnambs, 2020)—also in meta-analyses (Nuijten et al., 2015). Moreover, since small-studies yielding null effects are even less likely to published, this problem is further exacerbated (Egger et al., 1997; Stanley, 2017; Stanley & Doucouliagos, 2014). Thus, considering small-study effects is vital to make appropriate inferences from meta-analyses (see Gnambs, 2020). Robust Bayesian meta-analysis incorporates both selection models as well as the precision-effect test and precision-effect estimate with standard errors (PET-PEESE) method (Bartoš et al., 2022; Stanley, 2017; Stanley & Doucouliagos, 2014). This allows robust Bayesian meta-analysis to account for small-study effects in two complementary ways. First, selection models are a flexible and powerful class of methods to consider small-study effects. Selection models estimate the relative probability a given study is published based on its reported *p* value. Second, the PET-PEESE method models the relationship between effect sizes and standard errors across all included experiments. If standard errors strongly predict effect sizes, studies with smaller sample sizes are associated with larger effect sizes. In such a case, small-study effects are relatively likely to affect the overall meta-analytically estimated effect size.

Robust Bayesian meta-analyses provide Bayes factors for the presence or absence of a meta-analytic effect, heterogeneity (variance between studies) and publication bias (including small-study effects). The robust Bayesian meta-analysis method builds on the principle of Bayesian model averaging (Maier et al., 2023). In this approach, all different combinations of these models are fit: for example, Model A assumes the presence of a meta-analytic effect, but assumes heterogeneity and small-study effects are absent (or 0). In contrast, Model B assumes the presence of an effect and small-study effects but no heterogeneity. All possible different (in this case 36) models are fit. Then, these models are compared based on fits to the observed data (i.e., marginal likelihoods). Using Bayesian model averaging, these analyses provide Bayes factors (Morey & Rouder, 2011) to indicate the evidence for the alternative (indicated as BF_10_) as well as for the null (indicated as BF_01_) hypotheses for the meta-analytical effect of interest, heterogeneity (variance between studies) and small-study effects (Bartoš et al., 2022). Following Bartoš et al. (2022), we set the prior probability for publication bias-adjusted models to 0.5, and then divided this probability equally across selection models and PET-PEESE models. 

All code and data to reproduce the results are available online (https://osf.io/87v9q/).

## Results

### Meta-analyses

A generic inverse fixed-effects variance meta-analysis was run across all experiments. This yielded a significant but small selective RME effect, *d*_z_ = 0.16, 95% CIs [0.08, 0.25], *p* < .001, *k* = 14, *n* = 637. The *I*^2^ and Cochran *Q* tests suggested that heterogeneity was not problematic, *I*^2^ = 29.1%, *Q*(14) = 18.34, *p* = .15.

**Fig. 3:**
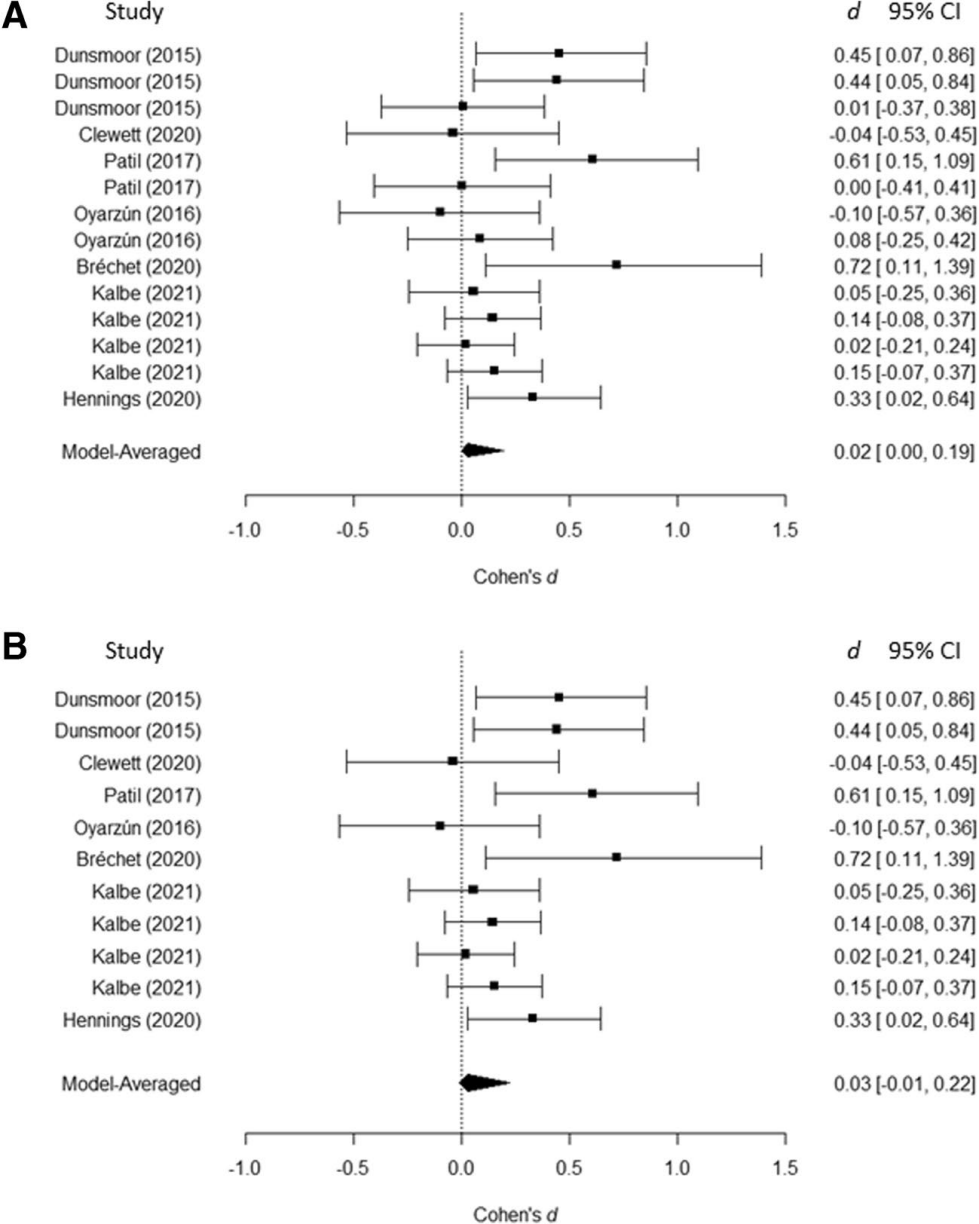
Forest plots of the robust Bayesian meta-analyses. Effect size is represented in Cohen’s *d*_z_ with 95% confidence intervals per individual experiment as well as collapsed across experiments. **A** Includes all experiments. **B** Includes only studies in which an interval between the study session and the surprise memory test was implemented of at least 1 hour

Next, a robust Bayesian meta-analysis incorporating the PET-PEESE and selection models methods was performed across all experiments (Fig. 3A). In contrast to the analysis above, this analysis yielded evidence in favor of the null hypothesis, BF_01_ = 3.32, *d*_z_ = 0.02 95% CI [0.00, 0.19]. This discrepancy was likely caused by small-study effects, BF_10_ = 11.39. The effect of small-study bias likely arises due to the relatively large samples from Kalbe and Schwabe (2021; *M* = 71.5, *SD* = 15.83), in which no significant selective RME effects were reported. In contrast, experiments showing significant effects had on average smaller samples (*M* = 28.8, *SD* = 7.9; Bréchet et al., 2020; Dunsmoor et al., 2015; Hennings et al., 2021; Patil et al., 2017). There was some evidence against heterogeneity, BF_01_ = 3.03. This can also be observed in Fig. 3A: Because across experiments, either a null effect or an effect in the positive direction is observed—no experiments show a strong negative effect.

Existing literature suggests that an interval between the studying session and the memory test is necessary in order for RME effects to occur (Dunsmoor et al., 2022). Such an interval allows for the influence of consolidation processes (Bréchet et al., 2020; Cowan et al., 2021; Dunsmoor et al., 2015; Squire et al., 2015). To test whether experiments with such an interval would show an effect, another robust Bayesian meta-analysis was run that included only experiments with such an interval (Fig. 3B). Again, support was provided for the null hypothesis, BF_01_ = 3.05, *d*_z_ = 0.03, 95% CI [−0.01, 0.22], and there was strong evidence for the influence of small-study effects, BF_10_ = 12.74. As in the previous analysis, no evidence was found for heterogeneity, BF_01_ = 2.68.

Finally, a robust Bayesian meta-analysis was conducted on only those experiments that did include both a consolidation interval and used electrical shocks as a salient event. This analysis was conducted since it is possible that the type of salient event could have affected the discrepancies between experiments. Again, no evidence was found for a selective RME effect, BF_01_ = 3.53, *d*_z_ = 0.02, 95% CI [−0.03, 0.21], and there was evidence for the influence of small-study effects, BF_10_ = 6.61. Also, no evidence was found for heterogeneity, BF_01_ = 3.10. This shows that even when only considering the eight experiments that used the most commonly used salient event, electrical shocks, no evidence was found for a selective RME.

In sum, the more traditional meta-analytical method shows evidence for a selective RME effect. In contrast, our Bayesian meta-analyses show that when taking into account small-study effects, the available published empirical studies in the literature do not give robust or strong evidence for a selective RME effect.

## Discussion

Since 2015, multiple studies have investigated whether salient events that occur after encoding of neutral information can enhance the memories of that neutral information in a semantically selective way (Dunsmoor et al., 2015). Findings regarding such selective RME effects have been equivocal thus far. Here, we conducted a meta-analysis to synthesize the available literature to assess the reliability of the selective RME effect.

The generic inverse variance fixed-effects meta-analysis provided support for a small but significant effect of selective RME. However, small-study effects, the tendency for smaller samples to be associated with larger effect sizes, can cause overestimations of effect sizes and lead to false positives—even in meta-analyses (Egger et al., 1997; Nuijten et al., 2015; Stanley, 2017). Thus, it is important to control for such effects when conducting meta-analyses (Bartoš et al., 2022; Gnambs, 2020; Maier et al., 2023). When considering the influence of small-study effects in the subsequent robust Bayesian meta-analyses, evidence for the null hypothesis was found. This suggests that this discrepancy between the two types of meta-analyses may have been caused by small-study effects. Thus, for those continuing to investigate this elusive effect, we recommend using relatively large samples to at least match the failed replication attempts of Kalbe and Schwabe (2021) (minimum of *n* = 80; a power analysis using the effect size, *d*_z_ = .16, from the generic inverse-variance meta-analysis (one-tailed paired-samples *t* test with .80 power) suggests a sample of *n* = 243). Together, our meta-analyses cast serious doubt on the reliability of the selective RME effect and find evidence for an influence of small-study effects.

Are there methodological differences between studies that could explain the discrepancies in results? Based on our literature search (see Table S1), methodological factors do not seem to substantially differ between experiments that did, or did not, find significant selective RME effects. This especially holds for the replication attempts reported by Kalbe and Schwabe (2021). In these four experiments, the authors took care to use a highly similar design to the original selective RME study by Dunsmoor et al. (2015) but with substantially larger sample sizes—with Experiment 4 being a nearly exact replication attempt. Only the duration of the interval between the incidental encoding and salient event phases seems to differ somewhat between the experiments by Kalbe and Schwabe (2021) and other experiments (10–20 minutes vs. ~5–6 minutes, respectively)—note that 4/15 experiments did not report this interval at all (Table S1). However, following general RME findings in humans and animals, this interval should be appropriate in every study, since the lifetime of the neutral tags is thought to last 0.5–3 hours, and thus this is never exceeded in the considered experiments (Dunsmoor et al., 2022; Redondo & Morris, 2011). Taken together, methodological factors do not seem to drive the differences between experiments that did, or did not, find significant selective RME effects.

Across the selective RME literature as a whole, one methodological factor should be (re)considered. It is possible that the initially neutral information may actually not have been encoded weakly, but instead relatively strongly. In order to find support for the behavioral tagging hypothesis, it is paramount that the initial (and incidental) encoding of the neutral material should be relatively weak. Otherwise it will be impossible for RME effects to occur (Dunsmoor et al., 2015, 2022; Viola et al., 2014). One experiment from Dunsmoor et al. (2015)—which was not included in the meta-analysis—showed this directly by inducing strong encoding by presenting items repeatedly. When items were initially already encoded strongly, no selective RME effect was found. Almost all included experiments (11 out of 14) used an immediate categorization task in which participants indicated whether a stimulus was an animal or a tool (see Table S1). The two other experiments from Patil et al. (2017) employed a delayed match-to-sample task in which participants matched items to one of two options after a brief interval. In the study by Bréchet et al. (2020), it is harder to classify whether the task during encoding was deep or shallow. Here, participants followed a stimulus flying through a virtual environment which passed along later relevant objects by pointing towards it. Most of the employed tasks during the encoding phase can be considered as instances of relatively deep encoding (Craik & Tulving, 1975; Ovalle-Fresa et al., 2021), and as such may have initiated relatively strong encoding of the initial stimuli. This may greatly have reduced the potential for selective RME effects to occur. One possibility to circumvent this in future work is to let participants determine visual features of the material, similar to experiments using verbal stimuli (Craik & Tulving, 1975; Ovalle-Fresa et al., 2021). Such a task should result in relatively weak/shallow encoding, possibly making it more likely to detect potential (selective) RME effects.

What can the absence of selective RME tell us about the memory system? It is clear that selective RME would have provided us with a highly adaptive mechanism as it selectively boosts past information that has become salient, while forgetting irrelevant information (Dunsmoor et al., 2022). This would help avoid/repeat similar salient experiences in the future. Given that we find very limited evidence for selective RME, we briefly want to discuss here how general RME may serve functions that are beneficial for survival. It is important to consider that animal and human studies have shown that memories for initially neutral material can be boosted retroactively in a nonselective, general manner (Moncada et al., 2015; Ramirez Butavand et al., 2020; Redondo & Morris, 2011; Viola et al., 2014). Consolidatory processes thus seem to prioritize information in temporal proximity to a salient event (Dunsmoor et al., 2022), regardless of the semantic associations to selectively boost initially neutral material. This could be a “safe” or conservative strategy to retroactively capture initially mundane information after this becomes relevant (also see Kalbe & Schwabe, 2021). One benefit of the “safe” general strategy can be illustrated using an example from Dunsmoor et al. (2022): An animal is suddenly attacked by a hidden predator, but the animal manages to escape this encounter. To enhance the chance of survival (Nairne et al., 2007, 2008; Nairne & Pandeirada, 2008; Shohamy & Adcock, 2010), it would be beneficial for survival to avoid such a dangerous confrontation in the future. Indeed, boosting the memory of what happened before the animal was attacked, can allow the animal to learn to avoid specific situations. For example, it might learn that a specific location, perhaps within a specific context, could be dangerous. Boosting not only the most relevant information, but also the less salient information may be sufficient for avoidance learning—although this is not necessarily adaptive. One benefit of conservative retroactive boosting is that potentially relevant information is not missed because everything is boosted. In contrast, it is more likely that some relevant details could be missed when selectively and specifically retroactively boosting initially mundane information. This interpretation is compatible with the behavioral tagging hypothesis, in that selective RME is not assumed (Ballarini et al., 2009; Moncada et al., 2015; Moncada & Viola, 2007; Viola et al., 2014). Future efforts could empirically test this hypothesis more directly by comparing whether presenting salient events or not boosts memory for the preconditioning phase in general. Oyarzún et al. (2016) report preliminary data showing that this does not affect memory performance, but as the authors note this experiment is underpowered and a large sample is necessary to make a reliable between-subjects comparison.

We note three limitations of the current meta-analysis. First, throughout the literature authors have pointed toward potential neurocognitive factors that may moderate selective RME. Among these are memory confidence, source memory and item typicality (Clewett et al., 2020; Dunsmoor et al., 2015; Hennings et al., 2021; Kalbe & Schwabe, 2021). Since not much data are available for each of these factors in relation to selective RME, we choose to not include them in the current analysis despite the fact that these factors could influence selective RME. However, we believe that these factors did not strongly affect our interpretations here. First, when considering only high-confidence responses and pooling across all experiments from Kalbe and Schwabe (2021), a small but significant selective RME effect is reported for corrected recognition scores, but not when analyzing *d′*. Moreover, the Bayesian analyses reported in Kalbe and Schwabe (2021) on both corrected recognition scores and *d′* support the null hypothesis, which posits that no selective RME occurs even when only considering high-confidence items. These analyses do not provide robust evidence for a selective RME effect in high-confidence responses. Moreover, their experiments suggest that strict control over item typicality does not affect the presence of selective RME (but see Hennings et al., 2021). Second, most of the included experiment were conducted by only two research groups—with seven experiments including J. E. Dunsmoor as an author and another four experiments including F. Kalbe and L. Schwabe as authors. It is apparent that the Dunsmoor group was involved in the design of at least Experiment 4 reported in Kalbe and Schwabe (2021), which minimized differences in methodological factors and increases comparability between studies. Although this does not necessarily bias our findings, it is noteworthy that not many research groups have published findings regarding selective RME and this should be considered when interpreting this meta-analysis. Lastly, the current number of included experiments may be considered relatively low. Although a higher number of experiments would be preferable for statistical inference, the current sample size is comparable to other meta-analyses (i.e., Anderson et al., 2020; Newbury & Monaghan, 2019). The data is also relatively homogeneous, increasing the power to detect selective RME effects. To demonstrate this further, we conducted a post hoc power analysis to assess which effects we could still pick up with adequate power using a generic inverse fixed-effects meta-analysis. This analysis showed that when assuming low heterogeneity, that effects as small as Cohen’s *d* = .20 could be reliably detected (power = .81; k = 14; *n* = 637). This further illustrates that the current analysis is sufficiently powered and sensitive to potential selective RME effects.

Taken together, we report no reliable evidence for a selective RME effect in humans. Small-study effects have impact on the current selective RME literature, and it is important to consider these effects. The absence of a selective RME effect does not exclude the possibility of more general forms of RME. Potential employment of such a general RME strategy could improve avoidance-learning by creating richer and vivid memories of what occurred before salient events. At present, no evidence seems to exist for a selective RME effect, and we advise caution whenever interpreting available findings.

### Supplementary Information

ESM 1 (XLSX 13 kb)

ESM 2 (R 7 kb)

## Data Availability

All code and data required to reproduce the meta-analytic results and figures are available here: https://osf.io/87v9q/.
